# Casein kinase 1α has a non-redundant and dominant role within the CK1 family in melanoma progression

**DOI:** 10.1186/s12885-016-2643-0

**Published:** 2016-08-03

**Authors:** Tobias Sinnberg, Jun Wang, Birgit Sauer, Birgit Schittek

**Affiliations:** Department of Dermatology, Division of Dermatooncology, Eberhard-Karls-University Tübingen, Liebermeisterstr 25, D-72076 Tübingen, Germany

**Keywords:** CK1, Melanoma, Beta-catenin, p53

## Abstract

**Background:**

We previously identified CK1α as a novel tumor suppressor in melanoma and reported that the loss of CK1α leads to increased proliferation and invasive growth of melanoma cells by strong activation of the Wnt/β-catenin signaling pathway.

**Methods:**

In this study we analyzed expression and the functional effects of the dominantly expressed CK1- isoforms α, δ and ε in melanoma cells by quantitative real-time PCR, western blot and immunohistochemistry. We down-regulated CK1 kinase activity with isoform specific siRNAs and small molecule inhibitors. Vice versa we overexpressed the CK1 isoforms α, δ and ε using viral vectors and tested the biological effects on melanoma cell proliferation, migration and invasion.

**Results:**

We show that protein expression of all three CK1-isoforms is downregulated in metastatic melanoma cells compared to benign melanocytic cells. Furthermore, the CK1δ and ε isoforms are able to negatively regulate expression of each other, whereas CK1α expression is independently regulated in melanoma cells. Inhibition of the expression and activity of CK1δ or CK1ε by specific inhibitors or siRNAs had no significant effect on the growth and survival of metastatic melanoma cells. Moreover, the over-expression of CK1δ or CK1ε in melanoma cells failed to induce cell death and cell cycle arrest although p53 signaling was activated. This is in contrast to the effects of CK1α where up-regulated expression induces cell death and apoptosis in metastatic melanoma cells.

**Conclusion:**

These data indicate that CK1α has a dominant and non-redundant function in melanoma cells and that the CK1δ and ε isoforms are not substantially involved in melanoma progression.

**Electronic supplementary material:**

The online version of this article (doi:10.1186/s12885-016-2643-0) contains supplementary material, which is available to authorized users.

## Background

Malignant melanoma is the most aggressive form of skin cancer whose incidence still increases worldwide. Melanomas arise from the transformation of benign melanocytes or nevi which can develop into dysplastic lesions before progressing into primary melanomas that can further invade into the dermis and metastasize via hematogenous or lymphogenic routes to distant sites [1]. Initiation and progression of melanoma have been associated with activation of key signaling pathways involved in proliferation, survival and dissemination. These include the Ras/Raf/MEK/ERK (MAPK) and PI3K/AKT signaling pathways as well as the Wnt/beta-catenin signaling pathway [2].

Protein kinases play a central role in signal transduction. By reversible phosphorylation of its substrate proteins, they exert influence on their activity, localization and function and thus are involved in almost all cellular processes and functions. The casein kinases (CK) belong to the serine/threonine kinases that are involved in a variety of cellular processes. Isoforms of the casein kinase 1 (CK1) family have been shown to phosphorylate key regulatory molecules involved in cell cycle, transcription and translation, the structure of the cytoskeleton, cell-cell adhesion and in receptor-coupled signal transduction. CK1 isoforms are key regulators of several cellular growth and survival processes, including Wnt, Hedgehog and p53 signaling, cell cycle control, DNA repair and apoptosis [3, 4].

In humans, six CK1 isoforms exist (α, γ1, γ2, γ3, δ and ε) and several splice variants for CK1α, δ, ε and γ3 have been identified. All CK1 isoforms possess a highly conserved kinase domain, but differ in length and sequence of the N-terminal and especially the C-terminal non-catalytic domains. CK1α plays a role in the mitotic spindle formation during cell division and in DNA repair mechanisms and further participates in RNA metabolism [3, 4]. The CK1 isoforms δ and ε are known to be important regulators in the circadian rhythm of eukaryotic cells. CK1α regulates apoptotic signaling pathways, however, there seem to be cell type-specific differences. In addition to the involvement in apoptotic signaling pathways, the CK1 isoforms α, δ and ε have important regulatory functions in the Wnt/β-catenin signaling pathway and seems to act in a concerted manner [5, 6]. Dishevelled (Dvl) is a key component in the Wnt/β-catenin signaling pathway. Upon pathway activation by Wnts, Dvl becomes phosphorylated by CK1 δ/ε [7]. CK1α acts as a negative regulator of the the Wnt/β-catenin signaling pathway by acting as a priming kinase for β-catenin phosphorylation on Ser45 which is a pre-requisite for further phosphorylations by GSK3β at the Ser/Thr residues 33, 37 and 41 [6, 8]. Without this priming phosphorylation β-catenin is not degraded and gets stabilized. A down-regulation of CK1α thus leads - due to the lack of “priming” phosphorylation - to an accumulation of cytoplasmic β-catenin. Indeed, we could show in metastatic melanoma cells that CK1α is downregulated which correlated with increased β-catenin stability [9].

The tumor suppressor protein p53 as well as the p53 interacting proteins MDM2 and MDMX are substrates of the three CK1 isoforms CK1α, CK1δ and CK1ε. In different cell systems CK1α and CK1δ are described to regulate p53 activity by phosphorylation of p53 itself or the p53 interacting proteins MDM2 and MDMX [3, 4, 10, 11]. Furthermore, the activity of p53 correlates with CK1α and CK1δ expression under stress conditions which points to an autoregulatory loop between CK1 isoforms and p53 [10, 11].

Some evidence points to an altered expression or activity of different CK1 isoforms in tumor cells. Database analyses from tumor cell lines and tissues indicated that the CK1δ and CK1ε isoforms might be slightly overexpressed on RNA level in some tumor types including melanoma, whereas RNA expression of CK1α is more variable but low in melanoma [4]. The CK1γ1-3 isoforms seem to be rather low in different cancers types. Expression analysis of CK1α in melanoma datasets clearly revealed a reduction in mRNA expression during melanoma progression and we could confirm the reduction of CK1α expression in metastatic melanoma cells on RNA and protein level [4, 9]. However, expression of the other CK1 isoforms has not been systematically analyzed in melanoma cells until now. Furthermore, it is not known whether there is a functional redundancy of the CK1 isoforms in the regulation of cell survival and tumorigenesis since several substrates are shared within the CK1 family such as β-catenin in the canonical Wnt pathway and p53 or Mdm-2 in the p53 signaling pathway [3, 4].

To identify the role of the different CK1 isoforms during melanoma progression we analyzed in this study a) the expression of the CK1 isoforms in melanoma cells of different progression stages in vitro and in vivo, b) the reciprocal influence of CK1 isoform expression for the α, δ and ε family members and c) the functional effects of gene expression modulation of individual CK1-isoforms (alpha, delta and epsilon) on melanoma cell survival, proliferation, migration and invasion.

## Methods

### Cell culture

Human melanoma cell lines were cultured for this study in RPMI 1640 medium with 2 mM L-Glutamine and 10 % fetal bovine serum (FBS; Biochrom, Berlin, Germany), penicillin, and streptomycin. They were subcultured 1–2 times a week when they reached 80 % confluency using Trypsin/EDTA (0.05 %/0.02 %) for detachment [9, 12]. The melanoma cell lines Malme-3 M, MDAMB435, M14, UACC62, SKMel28 and A375 originated from the NCI60 cell panel of the National Cancer Institute (NCI-DCTD repository). The melanoma cell lines WM35, WM115, WM793, WM3734, WM266-4, WM1366, 1205 LU, and 451 LU were generously provided by M. Herlyn (Philadelphia, USA). SbCl2 and SKMel19 were provided by C. Garbe (Tübingen, Germany). SKMEL30 was obtained from the DSMZ (Braunschweig, Germany) and SKMel147 was a kind gift of M. Soengas (Madrid, Spain). Melanocytes, primary fibroblasts and keratinocytes were isolated from human foreskin as described previously [13–15]. All of the cell lines used in our study were authenticated by sequence analysis of defined genes.

### siRNA mediated CK1 knockdown

2.5 × 10^5^ melanoma cells in 6well cavities were transfected with 50 pmol siRNA using RNAiMAX (Invitrogen, Darmstadt, Germany) according to the manufacturers protocol. The following siRNAs were used: siCSNK1A1 sense gaauuugcgauguacuuaa-dTdT, siCSNK1A1 antisense uuaaguacaucgcaaauuc-dTdG; siCSNK1D sense ugaucagucgcaucgaaua-dTdT, siCSNK1D antisense uauucgaugcgacugauca-dTdT; siCSNK1E sense ccuccgaauucucaacaua-dTdT, siCSNK1E antisense uauguugagaauucggagg-dGdA; siNONSIL sense acaacauucauauagcugccccc, siNONSIL antisense gggggcagcuauaugaauguugu (all synthesized by biomers.net, Ulm, Germany)

### Overexpression of CK1α/ δ/ ε

Wild type CK1 isoform cDNA was amplified using the Human Multiple Tissue cDNA (MTC) Panel II (Clontech, Saint-Germain-en-Laye, France) and isoform specific primers. CK1 cDNAs were cloned into the inducible lentiviral vector PLVX-tight-PURO (Clontech) by using In-fusion-HD Liquid Kits (Clontech) according to the manufacturer’s protocol. Sanger-sequencing was performed for verification of the correct cloned cDNA. Lentiviral particles were produced in HEK293T cells using the second-generation packing and envelope plasmids pCMVΔR8.2 and pMD2.G. Cells were transduced with lentiviruses as described previously [16] and doxycycline inducible melanoma cells were generated according to the manufacturer’s instructions (Tet-on Advanced System, Clontech). For overexpression of CK1α the previously described adenovirus was used [9].

### Inhibitor and doxycycline treatments

Small molecules were dissolved in DMSO and treatments were carried out using the indicated concentrations with vehicle controls. The following substances were used: Pyrvinium pamoate (Sigma, Taufkirchen, Germany), IC261 (Sigma), D4476 (Sigma), PF670462 (Sigma). Doxycycline hyclate (Applichem, Darmstadt, Germany) was dissolved in ddH_2_O and used at the indicated concentrations.

### 4-Methylumbelliferyl heptanoate (MUH) viability assay

For the analysis of proliferation and survival of melanoma cells, 2.5x10^3^ cells were seeded into 96-well plates and cultured with the indicated inhibitors for the indicated periods of time. After washing of the cells with PBS, 100 μg/ml 4-methylumbelliferyl heptanoate (Sigma, Taufkirchen, Germany) in PBS were added and incubated for 1 h at 37 °C. Microplates were measured in a fluorescence microplate reader (Berthold, Bad Wildbad, Germany) with Ex355/Em460 nm in sixtuplicates. Dose–response curves were generated using GraphPad Prism version 6 (GraphPad Prism Software Inc.).

### Cell cycle assay

2 x10^5^ melanoma cells per 6-well cavity were seeded and either transfected using siRNA or treated with 4 μg/ ml doxycycline to induce the overexpression of CK1δ and ε or transduced with the adenovirus (CK1α overexpression). Cells were cultured for 48 h before permeabilization and fixation of the cells in 70 % ice-cold ethanol for at least 1 h. Then they were re-suspended in PBS with 100 μg/ml RNAseA (Applichem, Darmstadt, Germany) and 50 μg/ml propidium iodide (Sigma, Taufkirchen, Germany) and stained for 30 min. FACS analysis for the detection of the distribution of the cells in the each cell cycle phase was performed with a LSRII FACS (BD, Heidelberg, Germany) using the FACSDiva software.

### 3D Melanoma spheroid culture

2.5 × 10^3^ SKMel19 cells were cultured on 1.5 % noble agar (Difco/BD, Heidelberg, Germany) coated 96well plates to form spheroids within 3 days. For overexpression of CK1 isoforms either 2 μg/ml doxycycline were added on the second day or the medium was supplemented with the adenovirus. After 3 days spheroids were embedded into 1 mg/ml collagen I (Corning/BD, Heidelberg, Germany) diluted in complete growth medium and cultured for four more days. In case of treatment inhibitors were added to the medium. Daily microphotographs were taken and the area of the spheroids was measured using ImageJ and normalized to the size at day 0 after collagen embedding for the evaluation of tumor cell invasion into the collagen matrix. After 4 days spheroids were stained using 1 μM calcein-AM (Life technologies, Darmstadt, Germany) and 100 ng/ml propidium iodide (Sigma, Taufkirchen, Germany) for fluorescence live-dead staining of the melanoma cells. Fluorescence was detected with an Axiovert fluorescence microscope (Zeiss, Jena, Germany). Mean fluorescence intensities of the red channel were used to determine relative cell death induction.

### Quantitative PCR

Total RNA was extracted from cells using the NucleoSpin RNA kit (Machery-Nagel, Dueren, Germany). Complementary DNA was made out of 1 μg total RNA using SuperScript II reverse Transcriptase (Invitrogen, Darmstadt, Germany) according to the manufacturer’s protocol. Quantitative real-time PCR (qRT-PCR) was performed with the SYBR green mix LightCycler 480 (Roche, Mannheim Germany). The relative expression levels of CK1 isoforms were determined using the ∆∆Ct-method method with ACTINB or 18S rRNA as reference genes. The primer sequences were as follows: CSNK1A1 forward 5’-aatgttaaagcagaaagcagcac-3’ and reverse 5’-tcctcaattcatgcttagaaacc-3’. CSNK1D forward 5’-acaacgtcatggtgatggag-3’ and reverse 5’-gaatgtattcgatgcgactgat-3’. CSNK1E forward 5’-tgagtatgaggctgcacagg-3’ and reverse 5’-tcaaatggcacacttgtctgt-3’. CSNK1G1 forward 5’-ctgtgaccgaacatttactttga-3’ and reverse 5’-tgcacgtattccattcgaga-3’. CSNK1G2 forward 5’-gaccttcacgctcaagacg-3’ and reverse 5’-ccggtagattaggctcttggt-3’. CSNK1G3 forward 5’-tgcaacaatccaaaaaccagt-3’ and reverse 5’-ctgcaaggtgagctctcaaa-3’. ACTINB forward 5’-ttgttacaggaagtcccttgcc-3’ and reverse 5’-atgctatcacctcccctgtgtg-3’. 18S rRNA forward 5’-cggctaccacatccaaggaa-3’ and reverse 5’-gctggaattaccgcggct-3’.

### Western blot

Protein lysates (30 μg) were subjected to SDS-PAGE and semi-dry blotting onto PVDF membranes (Roche, Mannheim, Germany). The antibodies used were as follows: anti-CK1α (Santa Cruz Biot., Heidelberg, Germany), anti-CK1δ (Santa Cruz Biot.), anti-CK1ε (Santa Cruz Biot), anti-p53 (Santa Cruz Biot), anti-p21 (Cell Signalling, Heidelberg, Germany), anti-β-catenin (Cell Signalling), anti p-S45-β-catenin (Cell Signalling) anti-β-actin (Cell Signalling). HRP conjugated secondary antibodies were used (Cell Signalling and Santa Cruz) and ECL substrates for chemoluminiscent detection. Densitometric semi-quantification was done by normalizing the band intensities of the target protein to the signal of β-actin with Scion Image.

### Luciferase reporter assay

2.5 × 10^5^ melanoma cells were seeded into 6well plates and transfected with 2 μg Super8xTOPFlash 16 h porst seeding using ScreenFectA (Genaxxon, Ulm, Germany) as recommended by the manufacturer. Twenty-four hours later cells were reseeded into 96 well cavities and the expression of isoforms was induced by the addition of doxycycline or of the adenovirus for 48 h. Then cells were lysed with 50 μl of passive lysis buffer (Promega, Mannheim, Germany) and luciferase activity was analyzed using D-luciferin as a substrate (Sigma) in a TriStar luminometer (Berthold, Bad Wildbad, Germany).

### Immunofluorescence analysis of melanocytic biopsies

Nevi, primary and metastatic melanoma FFPE biopsies were sectioned, heat induced epitope retrieval (HIER) was performed using citrate buffer pH6 and the sections were stained using 1:100 rabbit anti-CK1α (Abcam ab 136052), 1:1000 mouse anti-CK1δ (Abcam ab85320) and 1:100 goat anti-CK1ε (Santa Cruz sc-6471). As secondary antibodies donkey anti-goat(Cy3), donkey anti-mouse(Cy2) and donkey anti-rabbit(Cy5) were used (all 1:250; JacksonImmunoResearch/Dianova, Hamburg, Germany) before staining the nuclei with 1 μg/ml DAPI (Sigma, Taufkirchen, Germany). Biopsies were microscopically analyzed using a confocal microscope system (Leica TCS SP2, Heidelberg, Germany) and the mean fluorescence intensity of representative cells was quantified using the Leica LCS software. For semi-quantification the mean fluorescent intensities of at least 30 cells per sample were background subtracted and presented as relative fluorescence units.

### Kinase assay (K-LISA)

A 23mer peptide containing the exon 3 phosphorylation sites of β-catenin was synthesized as previously described [9] and the NH_2_ terminus was labeled with biotin. Melanoma cells were lysed using passive lysis buffer (Promega, Mannheim, Germany), and 5 μg of the protein lysates were incubated in kinase buffer (Cell Signalling, Heidelberg, Germany) together with 10 μg of biotin-labeled peptide for 30 min at 37 °C in streptavidin-coated 96well plates (Life technologies, Darmstadt, Germany). Plates were washed with PBS-T and anti–phospho-Ser45-β-catenin antibody (Cell Signaling) was added (1:500). HRP-conjugated secondary antibody (Cell Signalling) was used to detect the phosphorylated substrate measuring TMB substrate (Cell Signalling) at 450 nm in a microplate reader (Berthold, Bad Wildbad, Germany).

### Migration and invasion assay

#### Skin reconstructs

Organotypic skin reconstructs were prepared as described previously [13, 17, 18]. SbCl2 melanoma cells were transfected with the indicated siRNAs 24 h before epidermal reconstruction. Ten days after air-lifting the model reconstructs were fixed, paraffine embedded, sectioned, and H&E staining revealed the invasive capacity after knockdown of CK1α.

### Boyden chamber experiments

Invasion was assayed using invasion chambers coated with or without Matrigel basement membrane matrix (BD Biocoat Matrigel invasion chambers, BD Biosciences, Heidelberg, Germany) as described previously [9, 16]. After incubation for 20 h at 37 °C the invaded cells were fixed and counted after cell staining with hematoxilin-eosin. The assays were performed in triplicates, six fields were counted per transwell filter and the invasion index was calculated according to the manufacturerer’s protocol.

### Real-time migration assay

The kinetics of cell migration was assayed using the xCELLigence Real-Time Cell Analyzer (RTCA DP; Roche). CIM-plate 16 wells used and 10,000cells were plated in each well using serum-free DMEM. The lower medium chamber contained DMEM with 10 % FCS. Cells were allowed to settle for 30 min at room temperature before being placed in the RTCA DP in a humidified incubator at 37 °C with 5 % CO_2_. Data were recorded every 15 min for 24 h. Plotted curves represent the averages from three independent measurements.

## Results

### Expression levels of the CK1- isoforms α, δ and ε are downregulated in metastatic melanoma cells in vivo

**Fig. 1:**
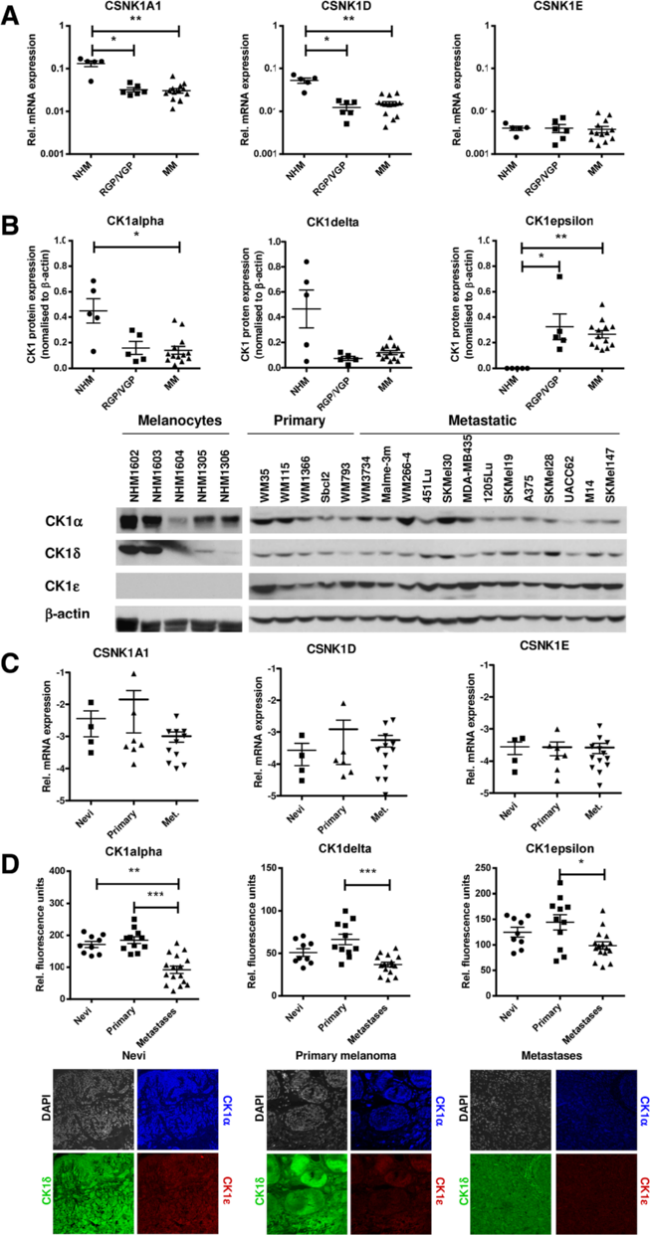
Expression of CK1 - isoforms during melanoma progression. **a** Relative mRNA expression (SYBR green real-time PCR) of three CK1 isoforms in melanocytic cells, namely normal human melanocytes (NHM), cell lines derived from primary radial growth phase (RGP) plus vertical growth phase melanoma (VGP) and cell lines from metastatic melanoma (MM). Normalized data (to ACTINB) are presented as scatter plot (mean with SEM). Kuskal-Wallis statistics with Dunn’s multiple comparison was used to test for significant differences (* *p* < 0.05; ** *p* < 0.01). **b** CK1α, δ and ε protein expression was determined by western blot analyses. Semi-quantification (ratios CK1/β-actin) are shown as scatter plots. Kuskal-Wallis statistics with Dunn’s multiple comparison was used to test for significant differences (* *p* < 0.05; ** *p* < 0.01). **c** Relative mRNA expression of three CK1- isoforms of patient-derived tissue samples. The analysis of CK-1 isoform expression was performed using benign melanocytic nevi (*n* = 4), primary malignant melanomas (*n* = 9), and metastatic melanoma (*n* = 13) by quantitative real-time PCR. Normalized data are presented as scatter plot (mean with SEM) and Kuskal-Wallis statistics with Dunn’s multiple comparison was used to test for significant differences (* *p* < 0.05; ** *p* < 0.01). **d** CK1α (*blue*), δ (*green*) and ε (*red*) expression in tissue sections of benign nevi (*n* = 11), primary melanomas (*n* = 11) or melanoma metastases (*n* = 16) was determined by immunofluorescence staining followed by confocal analysis. Kuskal-Wallis statistics with Dunn’s multiple comparison was used to test for significant differences (* *p* < 0.05; ** *p* < 0.01)

We analyzed expression of the CK1- isoforms α, δ and ε on RNA and protein level in normal human melanocytes (NHM) and melanoma cell lines representing the different progression stages in melanoma from radial growth phase (RGP), vertical growth phase (VGP) and metastatic melanoma (MM) (Fig. 1a-c). We found a consistent downregulation of CK1α expression on RNA and protein level in RGP, VGP and metastatic melanoma cell lines compared to NHMs. NHMs expressed significantly more CK1δ RNA compared to the melanoma cell lines. However, CK1δ protein expression was variable without significant differences in the analyzed melanoma cell lines. CK1ε expression was low in all cell lines analyzed and could not be detected in NHMs on protein level (Fig. 1a-c). CK1 γ1, γ2 and γ3 RNA expression was almost not detectable in the cell lines analyzed (Additional file 1: Figure S1A). Therefore, we focused in the following experiments on the CK1 isoforms α, δ and ε.

Next, we analyzed RNA and protein expression of the CK1 isoforms α, δ and ε in vivo in tissue samples of benign nevi, primary melanomas and metastatic melanomas using real-time PCR and immunofluorescence analyses, respectively. RNA expression of all three CK1 isoforms did not differ significantly in the different tissue types (Fig. 1c). By trend, CK1α RNA levels were reduced in preparations of metastatic melanoma. In contrast, on protein level we found a significant downregulation of all three CK1- isoforms in metastatic melanomas compared to primary melanoma cells (Fig. 1d). In summary, we found in melanoma cell lines in vitro and in melanoma cells in vivo a consistent downregulation of CK1α RNA and protein expression in metastatic melanoma cells. Furthermore, we detected a downregulation of CK1δ and ε protein expression in metastatic melanoma cells in vivo compared to primary melanoma cells. This did not correlate with RNA expression and with the expression levels of melanoma cells in vitro.

### CK1 δ and ε expression is partially reciprocally regulated by a posttranscriptional mechanism in melanoma cells

**Fig. 2:**
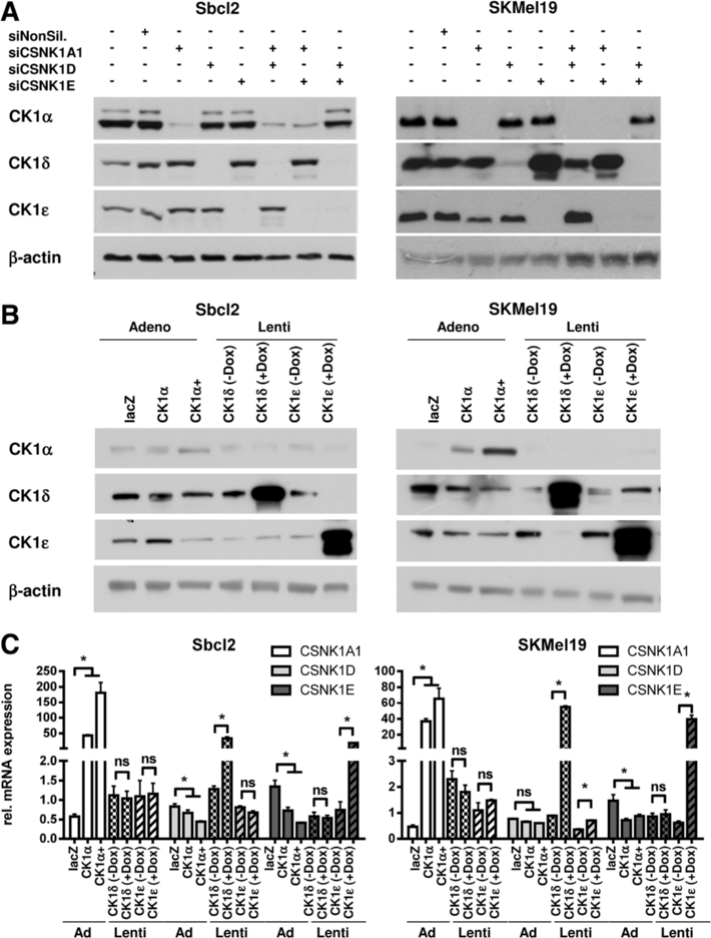
CK1δ and ε reciprocally regulate their expression by a post-transcriptional mechanism. **a** Specific siRNA mediated knockdown of CK1- isoforms in SbCl2 (left panel) and SKMEL19 (right panel) melanoma cells. The influence of the corresponding isoforms on the other two isoforms was evaluated by western blotting 48 h post siRNA transfection. Beta-actin detection served as a loading control. **b** Overexpression of CK1α, δ and ε in SbCl2 and SKMEL19 melanoma cells by viral transduction. Lysates were prepared 48 h after overexpression and western blots were probed with isoform specific antibodies and β-actin as a loading control. **c** Relative mRNA expression analysis of the three CK1 isoforms α, δ and ε after overexpression of the respective isoforms 48 h post induction/ transduction. 18S rRNA was used as reference gene. Ad5-LacZ transduced cells served as control for CK1α overexpression. Non-induced (Dox -) cells were used as control for overexpression of CK1δ and ε. All values were referenced to untreated SbCL2 and SKMEL19 control cells. Mutliple *t*-test was used to calculate statistically significant (* *p* < 0.05) expression differences after overexpression

So far it remains unknown whether the individual CK1 isoforms can regulate expression of the other isoforms in melanoma cells. Therefore, we downregulated expression of the CK1 isoforms α, δ or ε in the two human melanoma cell lines SbCl2 and SKMEL19 using isoform-specific siRNAs and analyzed RNA and protein expression of all three CK1 isoforms. As shown in Fig. 2a, downregulation of CK1α or CK1δ did not affect protein expression of the other isoforms in both cell lines. However, downregulation of CK1ε expression induced CK1δ expression most strongly in SKMEL19 cells (Fig. 2a). Combined inhibition of CK1α and CK1δ did only slightly affect CK1ε protein expression in SKMEL19 cells. However, downregulation of CK1α and CK1ε increased CK1δ protein expression, again most strongly in SKMEL19 cells. Downregulation of CK1δ and CK1ε had no effect on CK1α expression. These data suggest that CK1δ and ε regulate each other in a compensatory way and the expression is not or only mildly influenced by CK1α, whereas CK1α expression is independently regulated from CK1δ and ε.

To analyze whether overexpression of the specific isoforms resulted in similar effects we upregulated specifically CK1α expression by adenoviral gene transfer as previously reported [9] and CK1δ and CK1ε by a doxycycline-inducible lentiviral system in the two human melanoma cell lines SbCl2 and SKMEL19 (Fig. 2b). Overexpression of CK1α diminished only expression levels of CK1ε in SbCl2 and only at the highest induced expression level of CK1α. Induction of CK1δ reduced CK1ε protein levels in SKMel19 cells whereas elevated CK1ε levels were associated with lower CK1δ protein expression in SbCl2 cells (Fig. 2b). CK1α expression was not significantly affected by upregulation of the other CK1- isoforms. These data indicate that the δ and ε isoforms negatively regulate expression of each other. Analysis of RNA expression of the individual CK1 isoforms after induction of gene expression using real-time PCR indicated that overexpression of CK1α, CK1δ or CK1ε did not significantly influence RNA expression of the other CK1- isoforms (Fig. 2c). In summary, our data show that CK1δ and CK1ε negatively regulate expression of the respective other CK1 isoforms on a post-transcriptional level, whereas CK1α expression is not significantly affected by the other CK1- isoforms in melanoma cells.

### Modulation of CK1δ and CK1ε expression does not significantly influence melanoma cell viability and proliferation

**Fig. 3:**
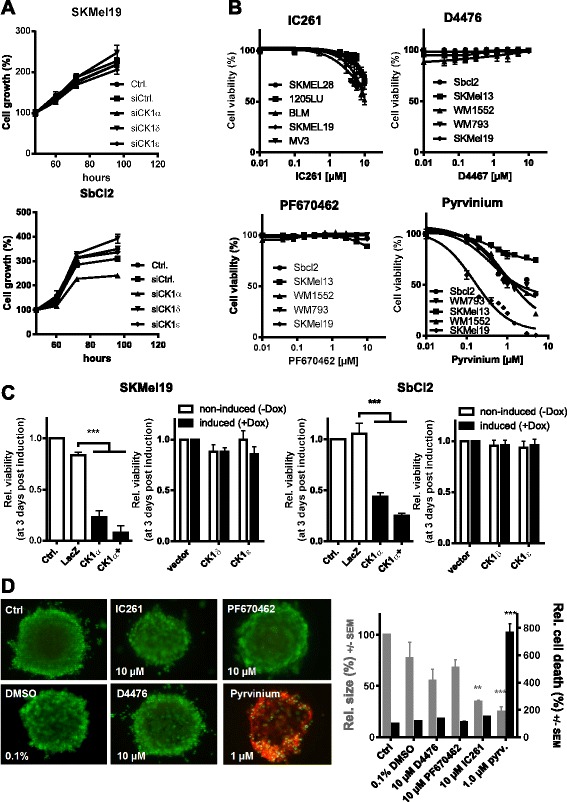
Modulation of CK1δ and CK1ε expression does not significantly influence melanoma cell viability and proliferation. **a** Inhibition of isoform specific CK1- activity via siRNA mediated knockdown of CK1α, CK1δ and CK1ε. SbCl2 (left diagram) and SKMEL19 (right diagram) cells were used and cell growth was monitored for 4 days using the MUH viability assay. Shown is the mean with SD of hexatuplicates. **b** Inhibition of CK1- activity via different small molecules (upper left and right plus lower left diagram) with predominant efficacy for CK1δ and CK1ε. Dose response curves using viability measurements (MUH assay) 72 h after treatment with the inhibitors are shown. Mean values with SD values of hexatuplicates are shown. The fourth diagram (lower right) shows dose response curves of melanoma cell lines treated with the allosteric CK1α activator pyrvinium at 72 h post start of treatment. **c** Effects of CK1 specific small molecules on 3D spheroid SKMel19 cultures. Spheroids were treated with the indicated concentrations of small molecules for CK1- inhibition or CK1α activation for 4 days. Live-dead staining with calcein-AM (1 μM) and propidium iodide (100 ng/ml) and size measurements are shown. Mean with SEM values of five spheroids are used. Multiple t-tests against vehicle controls were used for statistical analysis (* *p* < 0.05). **d** Effect of overexpression of the isoforms CK1α, CK1δ and CK1ε in SbCL2 and SKMEL19 melanoma cells. Isoforms were overexpressed as previously (Fig. 2b, c) and viability was assessed 72 h after overexpression of the respective CK1- isoforms by MUH assays. Shown are changes in viability after overexpression as mean values with SD of hexatuplicates are shown (*** *p* < 0.001)

Next, we looked for the functional effects of modulation of CK1- isoform specific gene expression on survival and proliferation of melanoma cells. First, we knocked down CK1α, CK1δ or CK1ε expression in SbCl2 and SKMEL19 melanoma cells using specific siRNAs (Fig. 2a). Ninety-six hours after transfection we analyzed survival and proliferation of the cells (Fig. 3a, Additional file 2: Figure S2A). In both cell lines the downregulation of CK1δ or CK1ε expression alone had no significant effect on cell growth or cell cycle. However, downregulation of CK1α expression retarded cell growth and increased the number of cell in the G1 phase of the cell cycle in SbCl2 melanoma cells, but not in SKMEL19 cells (Fig. 3a, Additional file 2: Figure S2A, B) confirming our previous study [9]. To further ascertain the effect of reduced CK1 activity on melanoma cell survival and proliferation we treated five different human melanoma cell lines with increasing doses of the CK1δ/CK1ε dominant inhibitors D4476 [19], PF670462 [20] or IC261 [21] and measured cell viability 72 h after treatment. As shown in Fig. 3b all three inhibitors did not significantly reduce melanoma cell viability. In a 3D spheroid culture model using collagen-embedded SKMEL19 spheroids similar results were obtained (Fig. 3c). At the highest concentration of IC261 a reduction in the size of the spheroids was observed which, however, was not accompanied with cell death induction (Fig. 3c). Only treatment of the cells with the CK1α activator pyrvinium resulted in propidium iodide positive dead cells (Fig. 3c). Also, overexpression of CK1δ or CK1ε in SbCl2 or SKMEL19 melanoma cells did not change melanoma cell viability and cell cycle (Fig. 3d, Additional file 2: Figure S2C). In contrast, activation of CK1α by pyrvinium [22] (Fig. 3b, c) or overexpression of CK1α in SbCl2 or SKMel19 melanoma cells (Fig. 3d) significantly reduced melanoma cell viability and induced apoptosis (Figs. 3b-d, Additional file 2: Figure S2C). These data indicate that CK1δ and CK1ε are not essential for melanoma cell survival and proliferation, whereas overexpression of CK1α reduces viability of melanoma cells. This suggests that CK1α is the most important CK1 isoform in melanoma cells with a non-redundant function in tumorigenesis.

### CK1α but not CK1δ and ε functionally affects melanoma cell migration and invasion

**Fig. 4:**
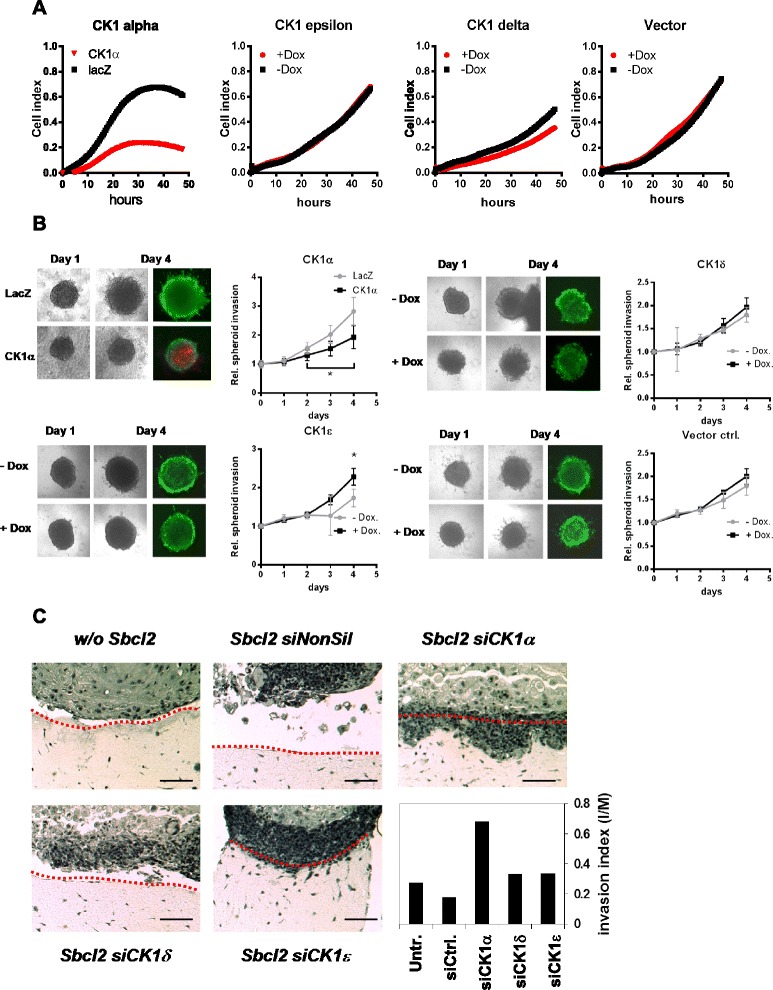
Functional effects of the modulation of CK1α, δ or ε on melanoma cell migration and invasion. **a** Real-time migration (upper panel) assays using the XCelligence DP analyzer. SKMEL19 Tet-On cells were induced to overexpress CK1- isoforms (red symbols) by doxycylcline pre-treatment for 48 h before seeding into the DP plates. Non-induced cells without doxycycline were used as reference controls (*black symbols*). For efficient overexpression of CK1α (red symbols) the adenoviral overexpression system was used 16 h before seeding the cells into the DP plates and effects were measured against lacZ control-transduced cells (*black symbols*). Shown are the cell indices of the measured impedance signals over 48 h. **b** SKMEL19 melanoma spheroid assay after CK1 overexpression (starting 24 h before collagen type I embedding). Spheroid spreading into the collagen matrix was microscopically monitored daily up to 4 days to estimate the invasive potential by referencing to day 0. Five spheroids were used for the calculations (Mean with SD; * *p* < 0.05). **c** Organotypic skin reconstructs with CK1 knockdown in SbCL2 melanoma cells. H&E staining is shown to reveal the invasive capacity into the dermal part after knockdown of CK1α. Matrigel coated invasion assays quantitatively show the invasive capacity of SbCl2 melanoma cells after knockdown of CK1 (lower right diagram)

In order to evaluate a further putative function of the CK1 isoforms in tumorigenesis - an increase in the migratory behavior of the tumor cells - we induced the expression of CK1α, δ and ε isoforms in SKMEL19 melanoma cells by doxycycline treatment and measured the migratory potential of the cells over time using the XCelligence system. Overexpression of CK1δ or ε in the melanoma cells led to no difference in the migratory behavior compared to the non-induced cells (Fig. 4a). However, overexpression of CK1α significantly decreased migration of the melanoma cells. 3D spheroid assays confirmed the results revealing no influence of the CK1- isoforms δ and ε on melanoma cell invasion of SKMEL19 cells into a collagen I matrix (Fig. 4b). CK1α overexpression significantly reduced the invasive growth within the monitored 4 days and again induced cell death. To further evaluate the effect of the CK1- isoforms on the invasive potential of melanoma cells we used an organotypic skin reconstruct using SbCL2 cells with siRNA mediated knockdown of the three CK1- isoforms which were seeded together with primary human keratinocytes as an epidermal layer. Since SbCL2 cells originate from an RGP melanoma they do not have the capacity to invade deep into the dermal part by breaking through the basal membrane which separates epidermal from dermal parts. Knockdown of CK1α resulted in a pro-invasive phenotype indicated by dermally invading melanoma cell nests as we showed before [9]. Knockdown of the other two CK1- isoforms δ or ε had no detectable effects on the growth characteristics in the skin reconstruct model (Fig. 4c). Our data indicate that CK1δ and ε do not affect survival and migration/invasion of melanoma cells in contrast to CK1α which seems to be the dominant active CK1- isoform in melanoma cells.

### CK1α, δ and ε differentially influence beta-catenin and p53/p21 signaling in melanoma cells

**Fig. 5:**
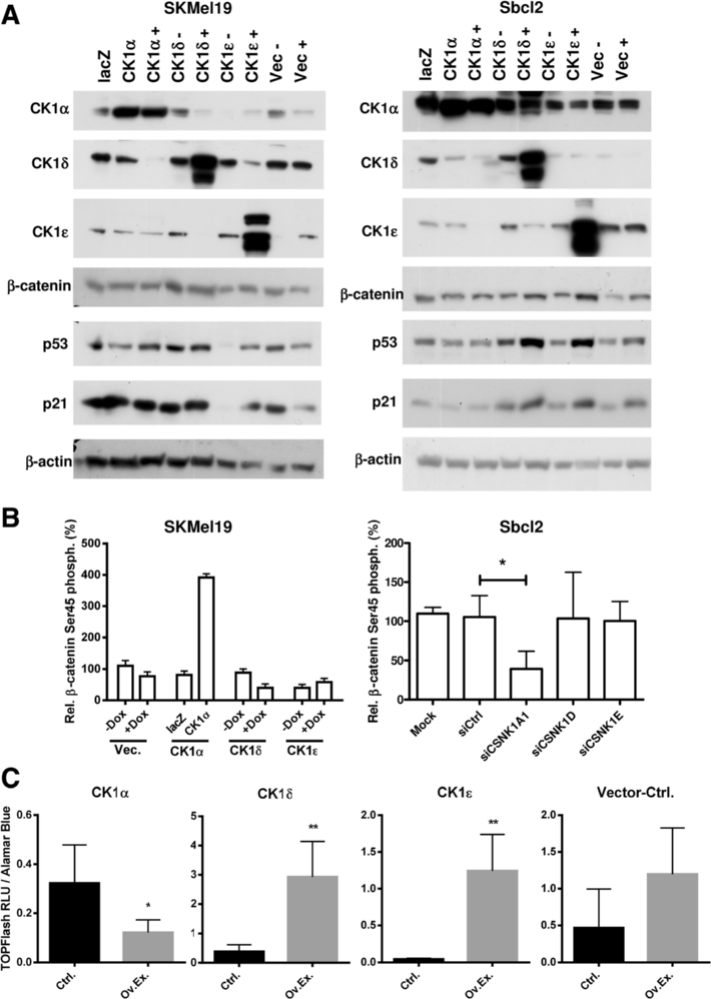
CK1α, δ and ε differentially influence beta-catenin and p53/p21 signaling in melanoma cells. **a** Western blotting of lysates from SbCl2 and SKMEL19 melanoma cells 48 h after overexpression of CK1- isoforms to detect the CK1- substrates β-catenin and p53 with its downstream target p21. **b** SbCL2 cells and SKMEL19 cells were used in a kinase assay using a peptidic β-catenin substrate (Ser45 phosphor-site) for quantitative determination of Ser45-specific kinase activity of the different isoforms. SKMEL19 cells overexpressing CK1 isoforms and SbCl2 cells transduced with siRNA 48 h before were lysed and subjected to a K-LISA CK1 assay using untreated cell lysates as reference. Biological triplicates were used in case of SKMEL19 samples and quadruplicates in case of SbCL2 cells to calculate the mean with SD (* *p* < 0.05). **c** Super8xTOPFlash reporter plasmid was transfected into SKMEL19 cells overexpressing CK1- isoforms and luciferase activity was measured in hexatuplicates for estimation of the TCF/LEF mediated and β-catenin dependent transcriptional activity. Luciferase activity was normalized to cell viability (* *p* < 0.05; ** *p* < 0.01)

It is known that β-catenin is a substrate of CK1α, δ and ε [3]. Whereas phosphorylation of β-catenin at Ser45 by CK1α results in degradation of β-catenin, CK1 δ/ε are involved in the activation of the Wnt/β-catenin pathway by the phosphorylation of dishevelled (Dvl). We analyzed whether overexpression of the individual CK1- isoforms as described above affects expression and activity of β-catenin signaling. Interestingly, β-catenin total protein levels did not change 1–2 days after CK1- isoform specific overexpression (Fig. 5a). However, as expected phosphorylation of Ser45 of β-catenin was increased after overexpression of CK1α (Additional file 3: Figure S3A) and this directly correlated with the influence of CK1α levels on the capacity to phosphorylate Ser45 in melanoma cells in a kinase assay (Fig. 5b). Overexpression of CK1α in SKMEL19 enhanced the kinase activity causing Ser45 phosphorylation, whereas the respective knockdown in SbCl2 decreased this activity. The other CK1- isoforms δ and ε did not show significant impact on the phosphorylation of Ser45 of β-catenin (Fig. 5b).

In order to measure the general effect of CK1- isoforms on the canonical Wnt-signaling pathway we used a firefly reporter system (Super8xTOPFlash) and tested the luciferase activity in lysates of SKMEL19 cells after induction of CK1- isoform specific overexpression. As expected, CK1α overexpression decreased the endogenous signaling activity, whereas CK1δ and ε enhanced the canonical Wnt signaling (Fig. 5c). Doxycycline treatment alone as a negative control moderately induced the reporter, however to a much lesser extent as with CK1δ or ε overexpression. These results confirm an inhibitory effect on Wnt/β-catenin signaling of CK1α and an activating effect of CK1 δ/ε in melanoma cells.

In addition, CK1α, δ and ε are known to influence activity of p53 signaling by specific phosphorylation. Overexpression of CK1δ and ε increased the protein levels of p53 and its target p21 in SbCl2 and SKMEL19 melanoma cells. In contrast, overexpression of CK1α did not influence p53 and p21 expression in this analysis (Fig. 5a). This indicates that p53 signaling is predominantly activated by CK1 δ/ε and not by CK1α in melanoma cells. However, knockdown of CK1α increased p21 expression (Additional file 3: Figure S3B). This goes in line with previous findings that MDM2 is a target of CK1α and CK1 δ/ε can phosphorylate p53 at N-terminal activating phosphor-sites [23].

## Discussion

Isoforms of the CK1 family have been shown to phosphorylate key regulatory molecules involved in cell cycle, transcription and translation, the structure of the cytoskeleton, cell-cell adhesion and in receptor-coupled signal transduction. Although they share highly conserved kinase domains, they differ significantly in the non-catalytic domains, suggesting that each isoform may play a specific role in regulating biological processes [3, 4]. CK1 family members share a substrate sequence consensus in which position n-3 is necessarily occupied by an acidic group or a phosphor-amino acid. This consensus is D/E X X S/T for unprimed substrates or S/T-PO4 X X S/T for primed targets. However also non-consensus substrates exist like β-catenin and NFAT-4 hinting at putative CK1- isoform specific functions [3, 4]. The expression as well as the functional relevance of each CK1- isoform in tumor cells and a possible functional redundancy have not been comparatively analyzes so far. We describe for the first time the expression of the dominantly expressed CK1- isoforms α, δ and ε in melanoma cells and their functional relevance in melanoma progression. We provide strong evidence for a non-redundant and dominant role of CK1α compared to the other CK1 isoforms in tumorigenesis supporting our previous hypotheses [9]. We show that CK1α dominantly influences proliferation, invasion and progression of melanoma cells, whereas CK1δ and CK1ε do not significantly influence melanoma cell survival, proliferation, migration and invasion in vitro. This was unexpected since all three CK1- isoforms have been described to play key roles in cell proliferation and in the control of signaling pathways known to be important in tumor cells.

CK1α can be found at the centrosomes, microtubule asters and the kinetochore [3, 4, 24] and plays a role in the mitotic spindle formation during cell division and in DNA repair mechanisms as well as in RNA metabolism [25, 26]. CK1δ is also involved in regulating cell cycle progression. It interacts with the spindle apparatus and regulates phosphorylation of α-, β- and γ − tubulin [27–29]. In addition, it was shown that checkpoint kinase 1 (Chk1) is able to interact and specifically phosphorylate CK1δ and by this regulate the kinase activity [30]. Furthermore, inactivating mutations in CK1δ are able to impair SV40-induced cellular transformation in vitro and mouse mammary carcinogenesis in vivo [31] strengthening the important function of CK1δ in cell proliferation. CK1ε is able to interact with mitochondrial proteins in ovarian cancer cells and by this increase growth and survival of the tumor cells [32]. Furthermore, in breast cancer cells CK1ε is a key regulator of cell proliferation by modulating protein synthesis. CK1ε is able to phosphorylate the translation factor 4E-BP1, thereby regulating cap-dependent translation [33]. In addition, fibrosarcomas seem to depend on CK1ε and knocking down other isoforms of CK1 was not effective at inducing growth arrest in these cells [34]. However, one study shows that re-expression of CK1α in a lung cancer cell line in which the expression of CK1α is also low causes reduced cell proliferation in vitro and tumor growth in vivo [35]. Another study shows that a pharmacological increase of CK1α protein significantly diminished melanoma cell migration [36]. Furthermore, it was shown that activation of CK1α by pyrvinium inhibits the proliferation of colon carcinoma cells through inhibition of the Wnt / beta-catenin signaling pathway [22].

Despite the important role of these CK1 isoforms in cell cycle regulation and progression in different tumor types CK1δ and ε seems to be functionally redundant in melanoma cells since we find no functional effect on cell cycle or tumor progression after modulation of their expression level in melanoma cells. In contrast, overexpression of CK1α induces cell cycle arrest and apoptosis in metastatic melanoma cells and inhibits migration and invasion, whereas downregulation of CK1α in radial growth phase melanoma cells induces invasive tumor growth with a slightly reduced proliferation rate confirming our previous results [9]. This implies that each CK1- isoform seems to have a unique function in promoting the integrity and proliferation of specific types of tumor cells.

In various cancer types CK1- isoforms are overexpressed. Especially the CK1δ and CK1ε isoforms are overexpressed in most tumor types compared to the respective benign tissues [4]. However, we found that during melanoma progression protein expression of the CK1- isoforms α, δ and ε is downregulated. This was consistently seen for CK1α in vitro and in vivo, whereas expression of the CK1 δ and ε isoforms are more heterogeneous as the in vitro and in vivo expression data are not consistent.

It was reported that CK1ε enhances the β-catenin-dependent proliferation in breast cancer [37] and a point mutation in CK1δ promotes the emergence of colorectal adenomas [38]. In contrast, a down-regulation of CK1δ and ε-isoforms in a variety of tumor cell lines of different origin induced cell cycle arrest and apoptosis. These effects are also Wnt/β-catenin-independent, but dependent of activated RAS and inactive p53 [4, 39, 40]. Furthermore, it was shown that impaired CK1δ activity attenuates SV40-induced cellular transformation in vitro and mouse mammary carcinogenesis in vivo [31]. We clearly show now in this study that in the different melanoma cell models these CK1- isoforms have no role in cell cycle progression and migratory and invasive melanoma growth. However, overexpression of CK1δ or CK1ε resulted in higher activity of the Wnt/β-catenin signaling pathway and an increased p53 activity, whereas CK1α overexpression inhibited Wnt/β-catenin signaling and p53 activity. However, the suppressive effect on p53 activity seems to depend on a gene dosage effect of CK1α. Furthermore we showed that in metastatic melanoma cells CK1α is downregulated resulting in higher transcriptional activity of the Wnt/beta-catenin signaling pathway confirming our previous study pointing out that CK1α is a tumor suppressor in melanoma cells [9]. It seems that depending on the molecular background and oncogene addictions in the tumor cells different CK1 isoforms have dominant roles in the respective tumor types.

It is known that the CK1- isoforms CK1α, CK1δ and CK1ε are capable to N-terminally phosphorylate the tumor suppressor protein p53 in vitro and in vivo. This leads to a reduced interaction of p53 with MDM2 and thus to a stabilization and activation of p53 [3, 4]. However, phosphorylation of MDM2 by CK1α, CK1δ and CK1ε can also promote p53 binding and degradation. Furthermore, CK1δ is known to phosphorylate MDM2 on other sites, which prevents the degradation of p53 [41]. In addition it could be shown that after genotoxic stress it comes to a transcriptional activation of CK1δ by p53 pointing out to an autoregulatory loop between these two proteins [3, 4]. Therefore, the outcome of CK1-kinase activation on p53 signaling has to be carefully analyzed in each tumor model.

The p53 signaling pathway seems to play a pivotal role in regulating CK1α activity. Our first description of invasive tumor growth due to knockdown of CK1α was substantiated by an ensuing work, which demonstrated the rapid invasive growth of transformed cells in the small intestine of mice when p53 is inactivated together with CK1α [42]. This suggests that loss of p53 in combination with loss of CK1α activity favors invasive tumor growth. Interestingly, p53 is a substrate of CK1α. Knockdown of CK1α induces p53 transcriptional activity by reducing the inhibitory effect of the MDM2 homologue MDMX for p53 [43]. It was further shown that CK1α plays a central role in mediating MDM2 control of p53 [11]. CK1α stimulates p53 under stress conditions probably by direct phosphorylation of p53 [10, 40]. Thereby, CK1α could be a cellular fine-tuning tool for the regulation of p53 activity, which is dependent on the gene dosage.

## Conclusions

We show that CK1α has a non-redundant and dominant role in melanoma progression. It has still to be determined which functional role the CK1δ and ε isoforms have in melanoma cells independent of cell cycle progression and migration/invasion. The ability of the CK1- isoforms to regulate several important signaling molecules modulated in different types of tumors point out that they might be suitable targets for clinical intervention also in melanoma therapy.

## Abbreviations

Chk1, checkpoint kinase 1; CK, casein kinase; Dvl, dishevelled; FBS, fetal bovine serum; HE, hematoxilin-eosin; MM, metastatic melanoma; MUH, methylumbelliferyl heptanoate; NHM, normal human melanocytes; PI, propidium iodide; RGP, radial growth pase; RTCA, real-time cell analyzer; VGP, vertical growth phase

## Additional files

Additional file 1: Figure S1. Expression of CK1γ isoforms in melanoma. (A) Relative mRNA expression of the γ1, γ2 and γ3 CK1 isoforms in melanocytic cells namely normal human melanocytes (NHM), cell lines derived from primary radial growth phase (RGP) plus vertical growth phase melanoma (VGP) and cell lines from metastatic melanoma (MM). The analysis of CK1 isoform expression was performed by quantitative SYBR green real-time PCR. Data were normalized to β-actin (ACTINB) and presented as scatter plot (mean with SEM). (B) Relative mRNA expression of the γ1, γ2 and γ3 CK1- isoforms of patient-derived tissue samples. The analysis of CK-1 isoform expression was performed using benign melanocytic nevi (*n* = 4), primary malignant melanomas (*n* = 9), and metastatic melanoma (*n* = 13) by quantitative real-time PCR. Data were normalized to β-actin (ACTINB). Data are presented as scatter plot (mean with SEM). (TIF 600 kb) Additional file 2: Figure S2. Influence of the modulation of CK1 isoform expression on cell viability and cell cycle. (A) Inhibition of isoform specific CK1-activity via combined siRNA mediated knockdown of CK1α, CK1δ and CK1ε. SbCl2 (left diagram) and SKMEL19 (right diagram) cells were transduced with isoform specific siRNA or a non-silencing control and cell growth was monitored for 4 days using the MUH viability assay. Fluorescence intensities were normalized (100 %) to the start point at 24 h post transfection of the siRNA. Shown is the mean with SD of hexatuplicates. (B) Cell cycle analysis after knockdown of CK1 isoforms in SbCl2 and SKMel19 melanoma cells. After ice-cold ethanol fixation melanoma tumor cells were stained with 50 μg/ml propidium iodide containing RNase in PBS for 30 min and analyzed in a LSRII flow cytometer (BD). (C) Cell cycle analysis at 48 h after induction of CK1 isoforms revealed a significant subG1 apoptotic population only after overexpression of CK1α. (TIF 701 kb) Additional file 3: Figure S3. Effect of the modulation of CK1α expression on p53 and β-catenin signaling. (A) Western blot of lysates from SKMel19 cells at 48 h post adenoviral overexpression of CK1α for the detection of CK1 isoforms, S45-phosphorylated β-catenin and p53/p21. (B) Western blots for the p53 target p21 of lysates from SbCl2 and SKMEL19 cells at 48 h post transfection with CK1 specific siRNAs. (TIF 2129 kb)
