# Supplementary figures and images for: Casein kinase 1α has a non-redundant and dominant role within the CK1 family in melanoma progression
Source: BMC Cancer. 2016 Aug 3;16:594. doi: 10.1186/s12885-016-2643-0 (PMC4973074; doi:10.1186/s12885-016-2643-0)

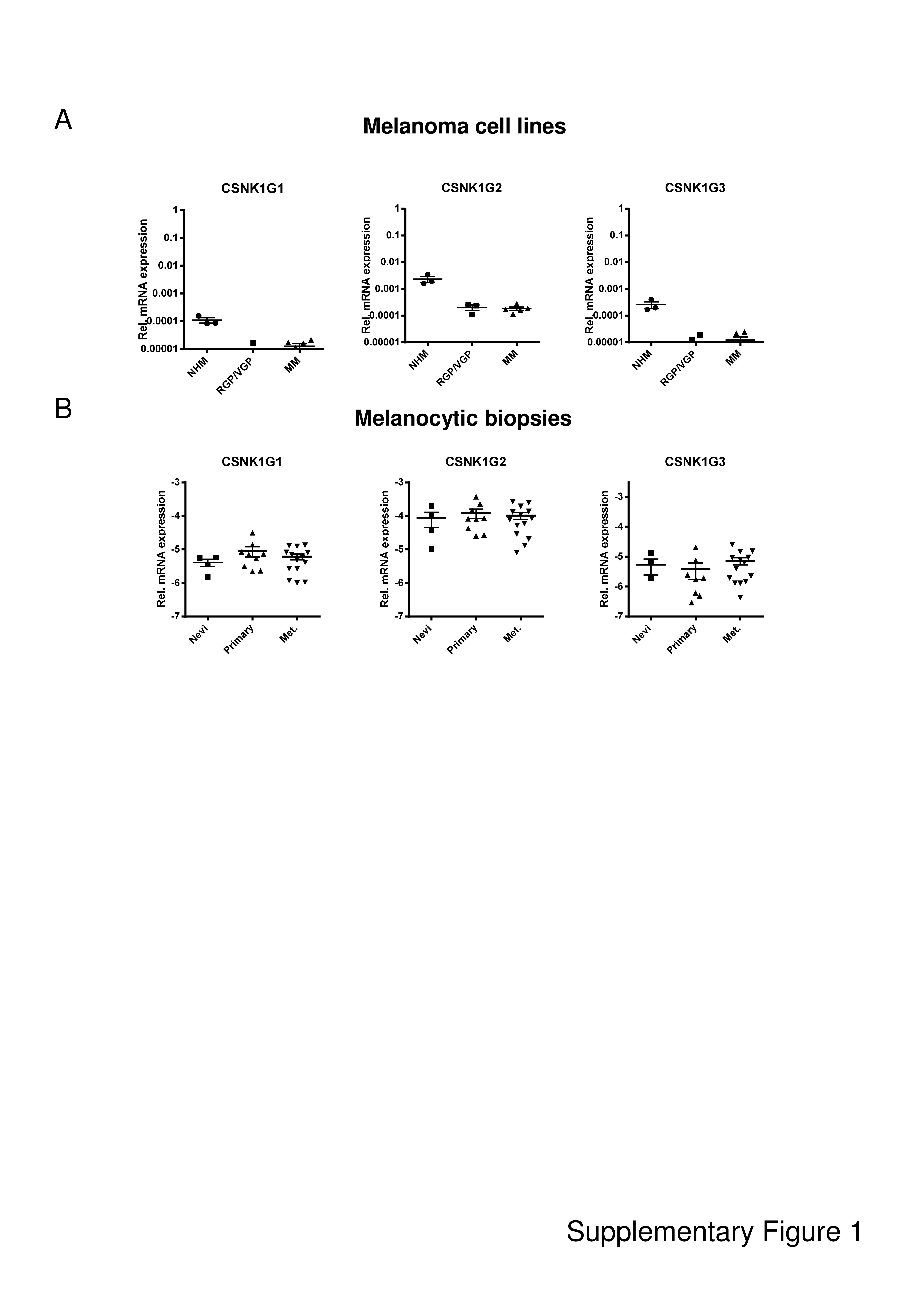

Supplement: Additional file 1: Figure S1. — Expression of CK1γ isoforms in melanoma. (A) Relative mRNA expression of the γ1, γ2 and γ3 CK1 isoforms in melanocytic cells namely normal human melanocytes (NHM), cell lines derived from primary radial growth phase (RGP) plus vertical growth phase melanoma (VGP) and cell lines from metastatic melanoma (MM). The analysis of CK1 isoform expression was performed by quantitative SYBR green real-time PCR. Data were normalized to β-actin (ACTINB) and presented as scatter plot (mean with SEM). (B) Relative mRNA expression of the γ1, γ2 and γ3 CK1- isoforms of patient-derived tissue samples. The analysis of CK-1 isoform expression was performed using benign melanocytic nevi (n = 4), primary malignant melanomas (n = 9), and metastatic melanoma (n = 13) by quantitative real-time PCR. Data were normalized to β-actin (ACTINB). Data are presented as scatter plot (mean with SEM). (TIF 600 kb) [file 12885_2016_2643_MOESM1_ESM.tif]

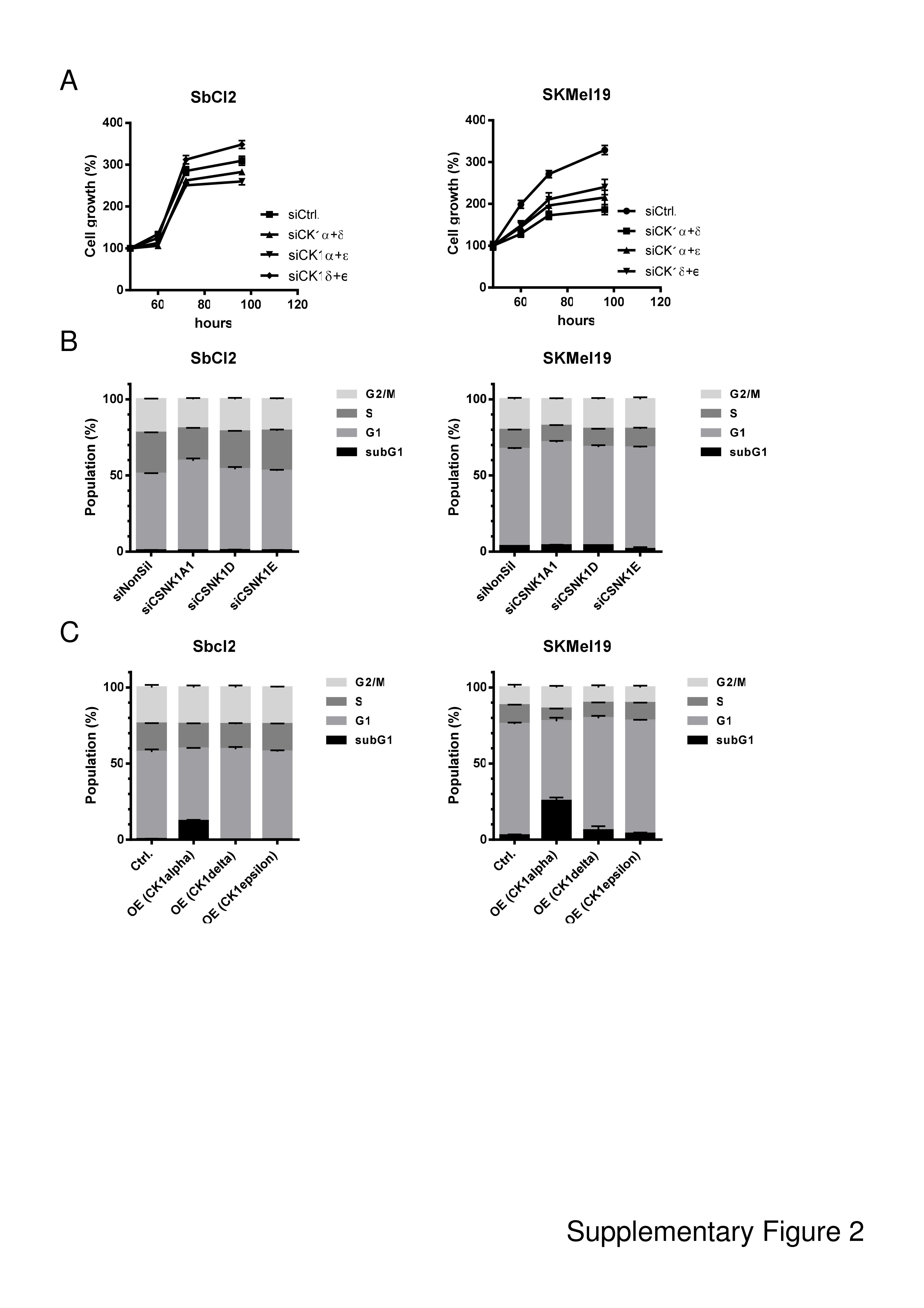

Supplement: Additional file 2: Figure S2. — Influence of the modulation of CK1 isoform expression on cell viability and cell cycle. (A) Inhibition of isoform specific CK1-activity via combined siRNA mediated knockdown of CK1α, CK1δ and CK1ε. SbCl2 (left diagram) and SKMEL19 (right diagram) cells were transduced with isoform specific siRNA or a non-silencing control and cell growth was monitored for 4 days using the MUH viability assay. Fluorescence intensities were normalized (100 %) to the start point at 24 h post transfection of the siRNA. Shown is the mean with SD of hexatuplicates. (B) Cell cycle analysis after knockdown of CK1 isoforms in SbCl2 and SKMel19 melanoma cells. After ice-cold ethanol fixation melanoma tumor cells were stained with 50 μg/ml propidium iodide containing RNase in PBS for 30 min and analyzed in a LSRII flow cytometer (BD). (C) Cell cycle analysis at 48 h after induction of CK1 isoforms revealed a significant subG1 apoptotic population only after overexpression of CK1α. (TIF 701 kb) [file 12885_2016_2643_MOESM2_ESM.tif]

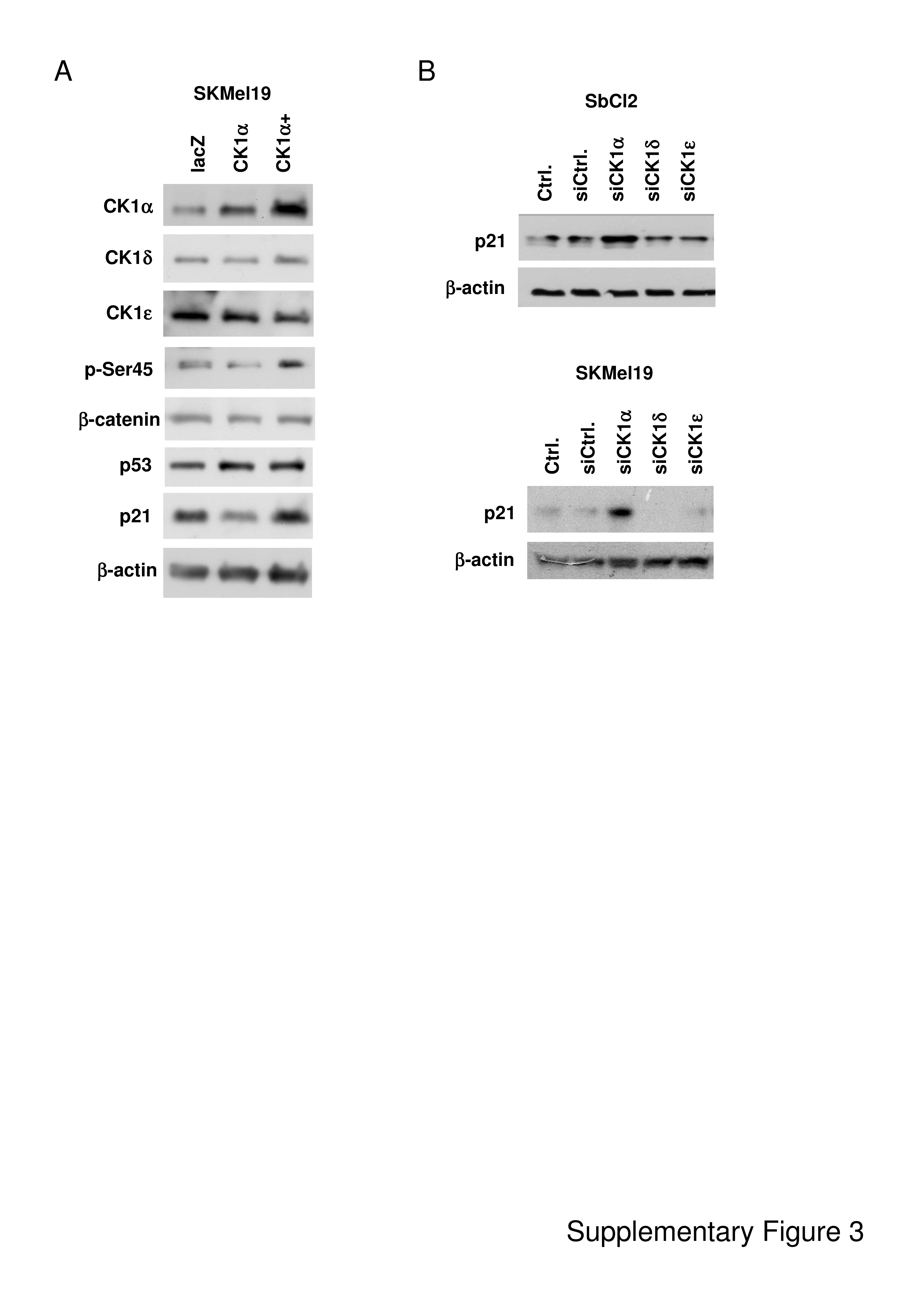

Supplement: Additional file 3: Figure S3. — Effect of the modulation of CK1α expression on p53 and β-catenin signaling. (A) Western blot of lysates from SKMel19 cells at 48 h post adenoviral overexpression of CK1α for the detection of CK1 isoforms, S45-phosphorylated β-catenin and p53/p21. (B) Western blots for the p53 target p21 of lysates from SbCl2 and SKMEL19 cells at 48 h post transfection with CK1 specific siRNAs. (TIF 2129 kb) [file 12885_2016_2643_MOESM3_ESM.tif]
